# Giving fruits and vegetables a tax break: lessons from a Dutch attempt

**DOI:** 10.1017/S1368980024000442

**Published:** 2024-02-15

**Authors:** Luc L Hagenaars, Tera L Fazzino, Joreintje Dingena Mackenbach

**Affiliations:** 1 Amsterdam UMC location University of Amsterdam, Department of Public and Occupational Health, P.O. Box 7057, Amsterdam 1007 MB, The Netherlands; 2 Philip R. Lee Institute for Health Policy Studies, University of California San Francisco, San Francisco, CA, USA; 3 University of Kansas, Department of Psychology, Lawrence, KS, USA; 4 University of Kansas, Cofrin Logan Center for Addiction Research and Treatment, Lawrence, KS, USA; 5 Amsterdam UMC location Vrije Universiteit Amsterdam, Epidemiology and Data Science, Boelelaan, Amsterdam, The Netherlands; 6 Upstream Team, Amsterdam, The Netherlands

**Keywords:** Taxation, Economic food environment, Vegetables, Fruit, Nutrition Policy

## Abstract

**Objective:**

Food taxation can improve diets by making unhealthy foods more expensive and by making healthy foods cheaper. In the Netherlands, a political window of opportunity arose in December 2021 to reduce the value-added tax (VAT) on fruits and vegetables to zero percent. The policy is now facing institutional friction along several fronts, however, delaying and potentially averting its implementation. We analysed this institutional friction to inform future food tax policies.

**Design:**

We qualitatively analysed open-access fiscal and health experts’ position papers about benefits and downsides of the zero-rate that were discussed with members of parliament in June 2023.

**Setting:**

The Netherlands.

**Participants:**

Not applicable.

**Results:**

Health and fiscal experts expressed noticeably different viewpoints towards the utility of the zero-rate. One important argument fiscal experts based their negative advice upon pertained to the legal restrictions for distinguishing between healthier and unhealthier forms of fruits and vegetables (i.e. the principle of neutrality). A zero-rate VAT on unhealthier forms of fruits and vegetables, e.g. processed cucumber, mixed with salt and sugar, would be undesirable, but differentiating between raw and processed cucumber would offend the neutrality principle.

**Conclusions:**

The Dutch attempt to give fruits and vegetables a tax break highlights the need for crystal-clear food classifications when designing food tax policies. Public health nutritionists should combine classifications based on caloric density, palatability, degree of processing and nutrient content to provide a database for evidence-informed tax differentiation according to food item healthfulness.

The low price of unhealthy foods relative to healthy foods is a major barrier to healthy diets^(1–3)^. Taxation policies are often suggested to reduce the cost differential. In line with this premise, many governments globally have adopted sugar-sweetened beverages taxes to increase the price of unhealthy sugar-sweetened beverages^(4,5)^. Using tax instruments to make healthy foods cheaper has been explored much less. In this short communication, we describe how the Dutch government announced a zero-rate value-added tax (VAT) on fruits and vegetables (FV) in December 2021^(6)^. Its implementation is now far from certain, however, after fiscal specialists questioned the measure’s effectiveness and feasibility. Our analysis, using position papers by fiscal and health specialists discussed in Dutch parliament in June 2023, finds that health and fiscal experts drew from different paradigms to inform their perspectives regarding the measure’s potential utility. We reflect on how public health nutritionists can take advantage of this case study by better understanding fiscal experts’ paradigms, which may promote more effective research and advocacy towards tax differentiating a broader range of (un)healthy food items. The Dutch attempt to give FV a tax break highlights that food healthiness classifications need more integration and dissemination to lawmakers who are on the front lines of policy decisions.

## Fruit and vegetables taxes in the Netherlands

At the time of writing, the Netherlands deploys two VAT rates: 9 % and 21 %. Most products and services fall under the VAT rate of 21 %. FV and other food items fall under the low VAT rate, which was raised from 6 % to 9 % in 2019. Implementing a lower VAT rate to promote healthier food access has been suggested by consumer organisations and FV interest groups in recent years^(7,8)^, e.g. after Dutch modelled studies suggested that cheaper FV could reduce healthcare costs and increase quality of life and productivity^(9)^. In 2020/2021, the Dutch government investigated options for a reduced VAT rate for FV, but EU Law (EU council directive 2006/112/EC) at the time only permitted a minimum VAT rate of 5 %^(10)^. Hence, VAT on FV would be reduced by only 4 % points. The Ministry of Finance doubted whether such a small price decrease would change consumption, whether it could clearly demarcate products to fall under the reduced VAT rate, and the tax office was reportedly only able to handle one reduced VAT rate (9 %)^(11)^. The idea was stalled, in the context of broader critique on the complicated nature of the Dutch tax system, outlined in a dedicated Ministry of Finance report^(12)^. Tax experts critiqued the many specialised tax categories and exemptions, which they attribute to the constant political tinkering with taxes for other purposes than raising revenue. Tax specialists saw VAT as inadequate for other purposes than raising revenue, due to the ‘principle of fiscal neutrality’: the notion that different VAT tariffs should not apply to products with a similar purpose^(12,13)^.

Early December 2021, the EU Council amended VAT regulations to enable member states to apply a 0 % VAT for FV and other items such as solar panels from April 2022 onwards^(14)^. This erased the problems that a 5 % VAT on FV was expected to decrease prices only marginally and that the tax office could not handle an extra tariff, since it already used a zero-rate at customs. On 15 December 2021, the new Dutch government’s coalition agreement was published, which included the sentence ‘*We bezien hoe we […] de BTW op groente en fruit naar 0 % kunnen verlagen’* (we will see how we can lower VAT on FV to 0 %) – under the condition that a thorough investigation of its feasibility and effectiveness would be conducted^(6)^. With polls suggesting 0 % VAT on FV was the most popular measure in the entire (fifty pages) coalition agreement^(15)^, it seemed only a matter of time before it would be implemented.

In March 2023, the research on feasibility and effectiveness of a zero-rate VAT on fruits and vegetables commissioned by the Ministry of Finance was published^(16)^. This research, conducted by an economic consulting group of tax specialists, advised not to pursue this policy. Arguments included that a zero-rate may not fully translate into lower consumer prices; that the limited price reduction would only lead to an estimated 4 % increased FV consumption; that the measure would not be cost-efficient due to high costs associated with implementation and enforcement and that its effects would be inequitable due to people with higher incomes consuming more FV. The experts expected litigation on the grounds that the measure may violate the principle of fiscal neutrality, giving the example of customers not differently using an unprocessed cucumber compared with cucumbers processed in a (side)dish. This negative advise was succeeded by a coordinated rebuttal of health scientists, local policy makers and health professionals, outlining why the zero-rate VAT would be advisable^(17)^, mostly by arguing that 4 % increased FV consumption is substantial on a population level, referring to Rose’s prevention paradox^(18)^.

In June 2023, Parliament organised a technical briefing with fiscal and health experts and interest groups, asking participants to outline their position in one pagers that are all published online (*n* 14), as is the briefing’s video recording^(19)^. JM and LH watched this recording, learning that fiscal and health experts disagreed about the feasibility and effectiveness of the zero-rate. JM and LH then coded specific arguments around the (un)feasibility and (in)effectiveness of the VAT in experts’ position papers (summarised in see online supplementary material, Supplement A) and thematically analysed these findings (Table 1). It is not yet known how the government weighed these arguments, since a decision on how to proceed was not yet made at the point of writing.

**Table 1:** Paradigmatic differences between fiscal and health experts towards the utility of implementing a zero-rate value-added tax on fruits and vegetables in The Netherlands

	Fiscal experts	Health experts
1	Attribution	Contribution
2	Reductionist	Holistic
3	Direct consumer effects	Systemic, delayed population effects
4	Household finances equity effect	Health equity effects
5	Pricing policy instruments	Pricing policies
6	Demarcation difficult due to VAT neutrality principle	Demarcation of lower VAT tariff political decision
7	Legally conservative	Legally negligent
8	Frustrated with feasibility and legal aspects not being taken seriously	Frustrated with healthy food policy not being taken seriously
9	Healthy government finances	Healthy population

## Paradigmatic differences between fiscal and health experts

All but one fiscal expert advised against the zero-rate, whilst all health experts spoke out in favour. Table 1 outlines our interpretation of differences between fiscal and health experts. First, the fiscal experts focussed on determining *if* the zero-rate VAT would *cause* a change in FV consumption (attribution). Some of the health experts, however, focused on whether the zero-rate VAT would *contribute to* changing in FV consumption. Second, the fiscal experts seemed interested in the isolated effect of the zero-rate VAT on food purchasing (reductionism). In contrast, health experts emphasised how a broad range of factors, including VAT rates, contribute to food purchasing. Third, the perceived low cost-effectiveness of the policy by fiscal experts seemed based on their focus on individual consumer behaviour. Health experts argued that the policy’s effectiveness should be determined by the size of the population consuming insufficient FV (75 % of the population), and that effects could be delayed, as markets adapt to a zero-rate. Fourth, the fiscal experts viewed the policy as economically regressive because people with higher incomes typically purchase more FV. In contrast, health experts emphasised the potential for improved health equity at the population level because people with lower incomes would be more price responsive. Fifth, fiscal experts highlighted their expertise on whether it is appropriate to use VAT as a policy action instrument. Health experts highlighted the more generic principle that pricing policies alter food purchasing. Sixth and seventh, while fiscal experts reported legally conservative views, health experts neglected legal aspects, arguing that VAT differentiation has always been a political decision. Eight and ninth refer to more deeply rooted frustrations. While fiscal experts were frustrated with the history of overtly complicated Dutch tax policies, health experts were frustrated with political inaction on diet-related disease prevention.

## Institutional friction towards food taxes

The above paradigmatic differences between fiscal and health experts, the history in which both frustrations are routed and the budget implications of implementing a zero-rate on FV (costing 500–950 million Euros per year^(16)^) have formed excessive ‘institutional friction’ towards an initially popular policy. Institutional friction concerns the observation within political science that policy adoption is often delayed by policymaking institutions –which can include different types of experts– and tend to act to maintain stability and incrementalism^(20)^.

A clear food healthiness classification system may decrease such institutional friction by aiding the feasibility of demarcating food items according to their healthfulness. Once this is in place, policymakers could go beyond only a zero-rate VAT on FV, ideally moving towards sophisticated tinkering with the prices of a broad range of food items to revert the current correlation between healthfulness and higher price^(21,22)^. The Dutch attempt to lowering FV VAT suggests that such a compelling healthy food taxation scheme would meet even higher levels of institutional friction, however, since there is no clear consensus about the most trustworthy (combinations of) food classification(s).

## Integrating healthy food classifications

Several classification approaches have been presented that categorise foods by caloric density, palatability, degree of processing or nutrient content, to identify foods that may increase disease risk, or be protective against disease. These classifications are described herein. Foods with elevated energy density (>2 kcal/serving size in g) have greater calories per bite and often contain added fats and/or refined carbohydrates that may increase caloric density^(23,24)^. Consumption of high energy density foods has been associated with increased obesity risk^(25)^. Foods that have low energy density contain more satiety promoting nutrients and lower calories per bite, which are often whole fruits and vegetables, and are associated with better diet quality and lower obesity rates^(26,27)^.

Hyper-palatable foods contain combinations of palatability-inducing nutrients (fat, sugar, Na and/or carbohydrates) at unnatural thresholds^(28)^. As a result, hyper-palatable foods provide a highly rewarding eating experience and delay physiological satiety responses^(29)^, explaining why hyper-palatable foods intake has been associated with overeating and obesity-related outcomes^(30,31)^. Foods that are not hyper-palatable typically contain one main palatability inducing nutrient (e.g. sugar) combined with satiety promoting ingredients (water, fibre), such as fresh, whole fruits and vegetables^(28)^.

The NOVA classification system identifies foods based on the nature and extent of their industrial processing. This four-tiered classification system identifies foods that range from minimally processed, which are typically edible parts of plants and animals derived directly from nature, to ultra-processed foods that undergo extensive industrial processing and/or contain industrial ingredients used to facilitate convenience in consumption and/or appeal^(32)^. Ultra-processed food intake has been strongly associated with disease, whereas consumption of minimally processed foods has been associated with lower obesity and metabolic disease risk^(33)^.

Other food classification schemes such as the European Nutri-Score^(34)^, Australian Health Star Rating^(35)^, Chilean nutrient profile model^(36)^ or the Dutch Choices logo^(37)^ are nutrient- or food-based schemes. Few studies have compared the performance of different classifications. One study in the Netherlands comparing adherence to the Dutch food-based dietary guidelines with the Nutri-Score and Choices classifications found significant discordance between the three schemes^(38)^. Slovenian^(39)^ and Australian^(40)^ comparative studies, however, concluded that the Health Star Rating and Nutri-Score were highly compliant ranking schemes.

Ideally, different classifications could be used in an integrated manner to identify foods that are minimally processed, that have not been modified to exaggerate their palatability and that are low in fat, salt and sugar and are nutrient dense. This premise would be consistent with the minimally processed NOVA definition that specifies edible parts of plants that occur directly in nature^(32)^, as well as low energy density foods that are typically described as containing satiety-promoting nutrients and are recommended in weight management interventions^(41,42)^. Additionally, evidence has indicated that the hyper-palatable foods definition has strong discriminant validity and (appropriately) does not identify fresh/raw fruits and vegetables as hyper-palatable^(28)^. Researchers therefore may consider creating a representative database of foods that are whole, fresh and healthy, categorised based on being minimally processed, not being hyper-palatable, having low energy density (<2 kcal/g) and having high nutritional value. A user-friendly, open source such database could facilitate public policy makers in clearly and evidence-supported differentiation of tax tariffs according to the healthfulness of food items.

### Conclusion

The Dutch government announced a zero-rate VAT on FV in December 2021, but whether FV will get their tax break remains uncertain at the point of writing. The institutional friction that this plan met nevertheless provides useful insight into how differently fiscal and health experts perceive the use of taxation for public health nutrition purposes. The case highlights how crystal-clear food classifications may inform FV definitions for designing FV taxation measures, and that integration between classifications is quintessential for exploring more compelling (un)healthy food taxation schemes. Combining classifications based on caloric density, palatability, degree of processing and nutrient content may provide a practical and evidence-supported database for determining which foods deserve a tax break and those that should make up for the lost revenue.

## Supporting information

Hagenaars et al. supplementary material Hagenaars et al. supplementary material

## Supplementary material

For supplementary material accompanying this paper visit https://doi.org/10.1017/S1368980024000442.
