# Supplementary material for: Giving fruits and vegetables a tax break: lessons from a Dutch attempt
Source: Public Health Nutr. 2024 Feb 15;27(1):e70. doi: 10.1017/S1368980024000442 (PMC10966848; doi:10.1017/S1368980024000442)
Supplement: Hagenaars et al. supplementary material [file S1368980024000442sup001.docx]

Supplement A. Summary of arguments included in position papers (N=13) submitted by fiscal and health experts to the hearing about the zero-rate Value-Added Tax (VAT) on fruits and vegetables (FV) in Dutch parliament.

| Position paper | Arguments opposing | | Arguments in favour | | Other arguments |
| --- | --- | --- | --- | --- | --- |
|  | Unfeasible | Ineffective | Feasible | Effective | e.g., fairness, loss of revenue |
| Belastingdienst (Tax Administration) | Problems demarcating FVs would require extra tax specialists which aren’t readily available, meaning the tax office cannot guarantee adequate enforcement.  Given the scope of products and financial interests involved, the Tax Administration foresees a considerable number of legal procedures opposing classifications of products falling outside the zero-rate. As this would mean a continuous discussion about what constitute FVs, the number of court cases might also keep increasing.  Due to the principle of neutrality, the zero-rate is unsuitable to promote the consumption of FV as this principle would be offended when products with similar usage among consumers would get a different VAT rate. Since this principle is leading, judges will also not take other policy goals (health promotion) into account. |  |  |  |  |
| GroentenFruit Huis (Fresh Produce Centre; an FV producer interest group) |  |  | If you make use of the international Global Product Classification, FVs can be defined easily. Principle of neutrality not relevant: consumers know the difference between a cucumber and cucumber spread. We can learn from other countries: Spain and Portugal have also lowered their VAT recently. | Zero-rate stimulates choosing the healthier option. In combination with other policies such as healthy school lunches it contributes to a healthier society.  4% is a lot on population level; 76 million kilos extra fruit and vegetables per year |  |
| Foodwatch (a consumer watchdog) |  |  | Principle of neutrality not relevant if the zero-rate applies to fresh, unprocessed fruits and vegetables. In Ireland, UK, Malta and Spain, the VAT rate for fruits and vegetables is also 0%. | Fiscal measures recommended by WHO and Farm-To-Fork strategy. The 4% increase in the SEO report is only a very rough estimate. But it would already be helpful. It would justify its implementation. Every extra carrot helps. |  |
| GGD GHOR (Regional Public Health Services Association) |  |  |  | A sugar tax will only be effective when coupled with policies that improve (economic) access to healthy food | It is unacceptable that some Dutch citizens cannot afford healthy food |
| Tax specialist mr A.J.A. Overwater |  |  | Controlling and maintaining the reduced tax is not an issue: there is no fiscal benefit to control and maintain the zero-rate VAT. In addition, there are already checks due to products falling under the low and high VAT rate.  Principle of neutrality is not an issue, because consumers know the difference between a raw cucumber and a cut and processed cucumber. | Reduced taxes are usually passed on to consumers. |  |
| Platform Voedsel en Gezondheid (Food and Health platform, a collaboration of applied science university professors working on healthy food consumption) |  |  | Feasibility can be increased by first applying the zero-rate VAT to unprocessed fresh and deep-frozen FV without added salt and sugar. This would include 90% of the revenue of FV.  The costs associated with implementing this policy will be mitigated by positive long-term health effects. | Higher consumption of FV is favourable for everybody because not only those with a low income consume too little FV.  The impact of a policy when it focuses on the whole population is also much larger than when it focuses on a narrow subpopulation (referring to Geoffrey Rose’s prevention paradox). |  |
| Dr. H Lelieveldt, associate professor of political science, University College Roosevelt, expertise in the food-climate-energy system. |  |  | A zero-rate VAT is one of the most feasible fiscal policies to promote healthier choices because it can be done at the national level.  The SEO report takes a narrow perspective on the principle of neutrality: the revised VAT guidance of the European Union requires that a reduced VAT rate ‘fits into the logic of the system of VAT rates’ and that ‘the benefits for society or the end user is clearly described’. |  | Signalling that the Dutch government supports making healthy choices easier is just as important as the actual effectiveness of the policy.  The zero-rate VAT will reduce tax revenue but how much exactly is uncertain. So, it will take political courage to prefer a difficult to quantity healthy benefit over the short-term reduction in tax revenue. |
| Alliantie Voeding voor de Gezonde Generatie (a health philanthropy collaboration aiming to healthily society for creating healthier generations) |  |  | A zero-rate VAT seems feasible because other European countries have also done this.  Deciding what products fall under the low and high rate is a political choice, rabbit food falls under the high rate and guinea pig food under the low rate. | Zero-rate VAT helps reduce the influx of new obesity cases.  Even a small increase in FV consumption helps prevent obesity.  More than a million Dutch living under the poverty rate will have better access to FV with a zero-rate VAT. | With the zero-rate VAT, the government would signal that they want to help citizens make healthy choices. |
| CPB (Netherlands Bureau for Economic Policy Analysis, an influential independent government research institute that provides economic policy analyses and projections) | High costs involved with dispute procedures related to the definition of FV. | High income groups profit more from the zero-rate because they purchase more FV.  Uncertain if and how consumers respond to price reduction. | Doctors are better able to estimate the health effects than tax specialists. | It is likely that the reduced rates will be passed on to consumers. | Don’t use VAT to influence consumer behaviour, high administrative costs among business owners who would have to differentiate more products |
| SEO Economisch Onderzoek (Economic consultancy firm that delivered the commissioned assessment of feasibility and effectiveness) | Due to the principle of neutrality, the zero-rate VAT on FV is unsuitable to promote the consumption of FV.  It is unlikely that the enforcement of the zero-rate will be sufficient, catering for high costs of or lack of implementation. | Effect of the zero-rate VAT is likely low because the price elasticity of FV is not very high. It is also uncertain if the price decrease will be passed on to consumers. SEO did not empirically investigate this. |  |  |  |
| Mr J. Bijl (fiscal lawyer) | For the principle of neutrality, it is not relevant whether consumers can distinguish between an unprocessed cucumber and a processed cucumber, but whether the use of these products is similar. An unprocessed cucumber is likely to be washed and cut before consumption, so the ultimate use of the product is similar as the processed cucumber.  In other countries, there have been court cases (UK, Germany, and Poland). |  | The discussion about defining FV is probably best settled through a focus on unprocessed FV. |  |  |
| Dr. F. Kreier (paediatrician expertise in obesity and neurology) |  |  |  |  | It is human nature to respond to environments that provide us with energy. Because losing weight is hard, it is best to prevent weight gain by focusing on stimulating healthy choices and discouraging less healthy choices. The zero-rate VAT on FV is crucial to do so. |
| Dr. C. Dijkstra (nutritional epidemiologist) |  |  | Definition of what is FV can be done based on the Dutch dietary guidelines, through a focus on fresh and unprocessed FV.  In Italy and Latvia, a reduced VAT on FV was successfully implemented and they provide a list of products defined as FV.  There have been no court cases in Latvia regarding the principle of neutrality. | SEO report did not study the actual effectiveness of a zero-rate VAT on consumption, and scientific evidence shows that price reductions are effective | VAT-policies are appropriate because they are universal preventative measures - in contrast to subsidies for specific subgroups.  The zero-rate VAT on FV would not be inequitable because those who experience a lower socioeconomic position are more sensitive to FV price decreases. |
| Dr. M. Poelman (associate professor consumption and healthy lifestyles) |  |  |  | Zero-rate VAT is effective to address the ***rise*** in unhealthy food consumption and obesity, not individual food consumption behaviours and disease risk. Also, the impact of a policy is not only dependent on its effect on behaviour, but also on the number of individuals displaying that behaviour. The impact of the zero-rate VAT therefore should not be based on its effectiveness alone (referring to Geoffrey Rose’s prevention paradox). |  |
